# Solving the clinker dilemma with hybrid output-based allocation

**DOI:** 10.1007/s10584-016-1884-x

**Published:** 2017-01-18

**Authors:** Frédéric Branger, Misato Sato

**Affiliations:** 1 CIRED, 45 bis, avenue de la Belle Gabrielle, 94736 Nogent-sur-Marne Cedex, France; 2 AgroParistech ENGREF, 19 avenue du Maine, 75732 Paris, Cédex France; 3 Centre for Climate Change Economics and Policy, LSE, Houghton Street, London, WC2A 2AE UK; 4 Grantham Research Institute on Climate Change and the Environment, LSE, Houghton Street, London, WC2A 2AE UK

**Keywords:** Carbon Price, Carbon Intensity, Emission Trade System, Carbon Leakage, Carbon Cost

## Abstract

Output-based allocation (OBA) is one of the main options discussed for addressing carbon leakage in emissions trading systems. This paper studies how different OBA designs affect incentives on mitigation and trade in the cement sector. To do so, we develop an analytical model of sector emissions as a function of technical parameters representing abatement levers. We propose a specific design called hybrid OBA, and show that unlike the alternatives, it provides incentives for firms to reduce the carbon intensity of production without offshoring production. We assess the feasibility of hybrid OBA through expert interviews and find that the main barriers identified, including technical and administrative complexities, are manageable. However, hybrid OBA represents a mid-term solution until more robust anti-leakage measures can be introduced, because of two key limitations of OBA in general - it does not provide incentives to reduce the consumption of cement or to accelerate the development of radical low-carbon technologies, both of which are necessary to deliver deep decarbonisation.

**Electronic supplementary material:**

The online version of this article (doi:10.1007/s10584-016-1884-x) contains supplementary material, which is available to authorized users.

## Introduction

An important concern around emissions trading systems (ETS) is the risk of carbon leakage. That is, the risk that companies facing high carbon prices will offshore parts of their production, together with emissions, to regions without equivalent carbon constraints. These risks are focused on a few key carbon intensive sectors such as steel and cement in which exposure to a high carbon prices severely increases production costs, and where the costs of carbon cannot be fully passed onto the consumer because of competition from imports (Sato et al. 2014). Failure to adequately address leakage not only implies adverse economic outcomes such as reduced industrial output and employment, but also reduced effectiveness of the ETS in containing CO _2_ emission levels globally.

To provide protection against carbon leakage, existing trading systems provide free allocation to leakage exposed sectors. Typically the number of free allowances is fixed *ex ante* and based on historic production volumes, as is the case in the EU ETS, the Kazakhstan ETS, the South Korea ETS, and Chinese pilot schemes.^1^ There is concern, however, that *ex ante* free allocation fails to provide robust leakage protection because it simply represents a lump-sum transfer. With an opportunity cost rationale, incentives under *ex ante* free allocation to sell unused free allowances and offshore production is similar to full auctioning. Experience with the EU ETS has also shown that free allocation based on historic production levels can lead to significant excess allocation during times of low economic activity, which results in weak incentives to drive down emissions (Neuhoff et al. 2014)^2^ and requires complex rules around new entrants, closure and partial cessation which can give rise to perverse incentives (Branger et al. 2015).^3^

To move towards more robust protection against potential leakage, broadly two options have been put forward. The first is output-based allocation (OBA) or *ex post* allocation, whereby free allocation is linked to actual or recent output levels. The literature has shown that OBA, compared to *ex ante* free allocation, ensures better the prevention of carbon leakage, suppresses excess allocation and gives rise to less adverse welfare impacts relative to other options (see Section 2). OBA has already been adopted in the New Zealand and California-Quebec ETSs. The second option is border levelling (or border carbon adjustments, BCAs) combined with full auctioning of allowances (Cramton and Kerr 2002; Grubb and Neuhoff 2006; Hepburn et al. 2006). BCAs have not yet been implemented in practice and many aspects need to be thoroughly investigated including legal, practical and political feasibility. Such effort will be worthwhile given auctioning has significant economic advantages compared to free allocation - it maximizes the incentive for all emission reduction levers (energy efficiency, fuel switching, breakthrough technologies and demand substitution) (Neuhoff et al. 2014), removes windfall profits, treats incumbent and new firms on an equal basis, and auction revenues can be used to reduce other distortionary taxes and improve macroeconomic efficiency.

This paper focuses on OBA and analyses how different designs of OBA affect mitigation and trade incentives in the cement sector. While the previous literature on *ex ante* allocation has shown how incentive distortions can arise from allocation rules (Neuhoff et al. 2006; Branger et al. 2015), this paper is the first to do so in the context of *ex post* allocation. One prominent issue with implementing OBA is the case of carbon-intensive intermediate goods (Quirion 2009); which in the cement sector translates into the so-called “clinker dilemma” (Demailly and Quirion 2006; Fischer and Fox 2012). 90 % to 95 % of CO _2_ emissions in the cement sector are due to the manufacturing of clinker, an intermediary product. Reducing the amount of clinker per tone of cement produced has been the main driver of CO _2_ abatement in this sector (Branger and Quirion 2015). The incentive to use less clinker is dampened, however, if allowances are distributed in proportion to clinker production (clinker OBA). Conversely, if allowances are distributed in proportion to the output of the downstream product cement (cement OBA), producers can save emissions (and sell allowances) by importing clinker instead of producing it, thereby causing carbon leakage.^4^ The clinker dilemma is not fully addressed in the current schemes using OBA.^5^ Yet the cement industry is the second largest manufacturing sector in terms of emissions,^6^ hence emissions at stake in the clinker dilemma are important, which makes a case for an adequate policy response to this issue.

This paper proposes an innovative and simple allowance distribution method called hybrid OBA to solve the clinker dilemma. Hybrid OBA is a clinker OBA modified with an allowances bonus-penalty depending on the clinker ratio (share of clinker in cement). Under hybrid OBA, the coverage of installations is also extended to downstream installations (grinding stations) which can play an important role in improving the carbon efficiency of cement but are currently excluded from most ETS policies. We develop an analytical model of cement emissions as a function of technical parameters representing abatement levers. We use empirical data to show that unlike alternative designs, the proposed hybrid method provides correct mitigation incentives - it encourages the reduction of clinker carbon intensity without encouraging the offshoring of clinker production. We also conducted interviews with both policy makers and industry executives in major cement companies in Europe, in order to guide the choices of implementation details.

The remainder of the paper is as follows. Section 2 reviews the existing literature on output-based allocation and summarises the key findings of both the predicted effects of OBA from analytical models, and the results of their numerical assessment in macro-economic modelling studies. In doing so, it clarifies the unique contribution of the microeconomic analysis in this paper. Section 3 sets the basis for the analysis by introducing the model of cement mitigation levers and defining the different OBA allocation designs, including the hybrid OBA. Section 4 then assesses the key advantages of hybrid OBA, and Section 5 discusses the possible implementation issues, using a case study of the EU ETS. Finally, Section 6 concludes.

## Literature review

The economic literature has documented the core economic mechanisms entailed by OBA. First, output is higher with OBA relative to full auctioning or *ex ante* free allocation. In the latter two options, firms have to purchase more (sell fewer) allowances with each additional output, whereas OBA grants more allowances for each additional output produced. Hence OBA acts as a production subsidy and diminishes the perceived carbon cost of home production, which can have adverse economic effects including on market entry (Fischer 2001). This may give rise to legal issues such as with WTO subsidy law (Rubini and Jegou 2012). Second, output price rises will be lower with OBA. Indeed, firms take account of the marginal revenue from the additional allowance they receive per unit of output. So the opportunity cost, which may be passed on consumers, corresponds to the difference between the firm’s performance and the benchmark, and not to the full amount of emissions as in *ex ante* allocations or full auctioning (Fischer 2001).

Third, consistent with higher domestic output and lower output prices, OBA protects the competitiveness of home energy-intensive industry and counteracts carbon leakage better than the two alternative options (Jensen and Rasmussen 2000; Bohringer and Lange 2005; Demailly and Quirion 2006; 2008; Fischer and Fox 2012). Fourth, with lower carbon cost pass through, consumers recieve limited economic incentive to curb consumption of carbon intensive products or switch to low-carbon alternatives, hence mitigation from demand substitution is largely forgone. Fifth, with output reduction lower than the social optimum, in the absence of other market failures, OBA raises the overall costs to meet a given emissions reductions target (Fischer 2001; Haites 2003). However, in the presence of market failures such as imperfect coverage (Bernard et al. 2007; Holland 2012), tax interactions (Goulder 2002) or imperfect competition (which calls for a carbon tax lower than the marginal damage, as pointed out by Buchanan (1969)), OBA (representing the combination of emissions pricing and production subsidy) may be welfare-enhancing in theory (Gersbach and Requate 2004; Fischer 2011).

Sixth, an advantage of OBA is that it avoids excess allocation because allocation is adjusted to output, thus suppressing over-allocation profits that may occur with *ex ante* allocation (Quirion 2009). Finally, OBA ensures incentives to adopt emission saving technologies are preserved when combined with benchmarks that are set at sufficiently ambitious levels, as the benchmarks become a focal point for energy and carbon efficiency improvements (Sterner and Muller 2008; Zetterberg 2014).^7^ At the same time, the incentive to develop new low carbon technologies and especially breakthrough technologies is limited compared to more efficient policies. Indeed there is less demand side pull because producers pay for and potentially pass on only the cost of emissions exceeding the benchmark rate, and not the full cost of emissions. In the case of cement, technologies reducing emissions per unit of output are still promoted but not alternative materials (like wood or innovative low-carbon cement not based on clinker).

These theoretical effects have been assessed with numerical models (typically partial equilibrium or macroeconomic models such as computable equilibrium model, or CGE) and provide several insights. First, the effect of the implicit subsidy can have a significant impact on the allowance price. Fischer and Fox (2007) find that in the US context, the allowance price is considerably higher (44 %) under OBA applied to all sectors compared to the other scenarios. The allowance price increase is even higher in Golombek et al. (2013) who focus on the European electricity sector with an extensive numerical model.

Second, OBA bears much less distributional issues than BCAs at the international level (Böhringer et al. 2012), because they do not provide foreign competitors the incentive to improve the carbon efficiency of production (Fischer and Fox 2012). However, distributional issues within the abating region are important if OBA does not apply to all sectors. Fischer and Fox (2007) find that the emissions reduction burden under OBA shifts from historical emitters in heavy industry towards other sectors such as agriculture, construction and final demand. Third, OBA is outperformed by economy-wide border carbon adjustments in terms of carbon leakage according to most CGE model studies (Böhringer et al. 2012; Böhringer et al. 2014). However, some models incorporating market failures show that combining auctioning with OBA targeted to energy intensive sectors may be more cost-effective than auctioning alone. This result is obtained by Lennox and Van Nieuwkoop (2010) for New Zealand and in the US context by Fischer and Fox (2010), but is not reproduced using the same model for Japan (Takeda et al. 2014).

Finally, two models with a more detailed representation of the cement sector give additional insights. Fowlie et al. (2012) develop a dynamic model of the US cement industry incorporating oligopolistic competition and leakage (imports are introduced through a competitive fringe). The comparison of four policies (grandfathering, auctioning, BCAs and OBA) regarding different exogenous social carbon costs shows that in terms of welfare, OBA is the least-worst (but still negative) policy for carbon prices under 45 dollars (being dominated by BCAs otherwise), mostly because other policies induce divestiture and exit leading to increased concentration of the industry. Meunier et al. (2014) build a model that incorporates existing capacities and demand uncertainty, and find that the optimal rate (in allowances per ton of clinker produced) for OBA would be almost three times lower than the actual one in Europe.

Overall, there is consensus that OBA, targeted only to energy-intensive industries, represents an attractive option as a mechanism to tackle carbon leakage concerns. However, the overall costs and distributional effects are such that it is likely to be a transitory compensation measure until a more permanent and efficient solution is put in place to address the priorities for emissions trading i.e. efficiency, equity and effectiveness (carbon leakage). Quirion (2009) and Heilmayr and Bradbury (2011) suggest that in the longer run, it is socially efficient that significant mitigation comes from the industrial sectors because marginal abatement costs tend to be higher in transport, agriculture and other sectors.

To our knowledge no papers have used microeconomic analysis to examine the incentive effects arising from different OBA allocation designs. The level of sectoral representation necessary precludes this type of analysis being conducted in numerical models discussed above. This paper therefore complements the existing literature by examining the detailed design of OBA and specifically a hybrid OBA for the cement sector.

## Modelling allocation to the cement sector

### The cement manufacturing process and abatement levers

Cement manufacturing can be divided in two main steps. First, clinker is produced by the calcination of limestone in a rotating kiln. The chemical reaction itself releases CO _2_ (around 0.53 tCO _2_ per ton of clinker) which are called process emissions and cannot be reduced.^8^ Clinker production accounts for over 90 % of CO _2_ in cement, two-thirds of which are process emissions. The remaining CO _2_ comes from the fossil fuels combusted to heat the kiln. The second stage is the blending and grinding of clinker with other materials to produce cement.

The two broad options to decrease cement carbon intensity are (i) decreasing the carbon intensity of clinker production^9^ and (ii) reducing the “clinker ratio” or proportion of clinker in cement, by substituting clinker with low-carbon alternative constituents of cement such as blast furnace slag or fly ash.^10^ Producers can also reduce their own emissions locally by offshoring clinker production, that is making cement with imported clinker, causing carbon leakage.

### Possible OBA allocation rules: cement, clinker or hybrid

Output-based allocation means that allocation is proportional to output. In the cement industry, clinker is the intermediary product to manufacture cement, so the allocation formula can be based on either clinker output (clinker OBA) or cement output (cement OBA).

For cement OBA, allocations are equal to the cement production (*Q*_*C*_) multiplied by a benchmark of cement carbon intensity (*B*_*C*_):

1 $$ A_{C}=B_{C} \times Q_{C}  $$

For clinker OBA, allocations are equal to the clinker production (*Q*_*K*_) multiplied by a benchmark of clinker carbon intensity (*B*_*K*_):

2 $$ A_{K}=B_{K} \times Q_{K} $$

In hybrid OBA, we modify the formula of clinker OBA with an additional term:

3 $$ A_{Hyb}=B_{K} \times Q_{K} + B_{K}(B_{R}-R)\times Q_{C}  $$

The additional term acts as an allowances bonus-penalty depending on the clinker ratio (*R*). When the clinker ratio is *lower* than a clinker ratio benchmark (*B*_*R*_), the additional term is positive, which means that *more* allowances are granted compared to clinker OBA. Conversely, when the clinker ratio is *higher* than the clinker ratio benchmark, *less* allowances are granted compared to clinker OBA. In terms of magnitude, the bonus-penalty term is designed so that, for a given cement production, a reduction of the clinker ratio brings a bonus corresponding to the avoided emissions of clinker manufacturing, if avoided clinker manufacturing was produced at the benchmark level of carbon intensity *B*_*K*_.

As we will see, this allocation method gives correct incentives in terms of mitigation and trade. Our hybrid OBA design also includes downstream installations (grinding plants) which are currently outside the scope of many trading schemes hence the scope of mitigation is expanded. How this equation differs from the California-Quebec and New Zealand allocation rules for cement is discussed in the Electronic Supplementary Material.

### Analytical framework

Modelling allocation methods typically uses a generic abatement function, and allocation enters the profit function as a simple function of historical output or actual output (e.g. Demailly and Quirion (2006) and Fischer (2001)). To our knowledge, technical parameters representing abatement levers have never been modelled when studying allocation methods. Our contribution is thus to assess precisely the impact that changes in the technical parameters have on carbon costs. To do so, we first express cement emissions as a function of variables of interest, and separate out the different levers of abatement. Notations and definition of variables are summarised in Table 1.

**Table 1:** Variables

Notation	Definition
*B*_*C*_	Benchmark for cement carbon intensity
*B*_*K*_	Benchmark for clinker carbon intensity
*B*_*R*_	Benchmark for clinker ratio
*I*_*K*_	Actual clinker carbon intensity of the plant
*R*	Actual clinker ratio of the plant ($\frac {{Q_{K}^{H}}+{Q_{K}^{I}}}{Q_{C}}$)
*Q*_*C*_	Cement produced on site
	and released onto the market
*Q*_*K*_	Clinker produced on site (${Q_{K}^{H}}+{Q_{K}^{O}}$)
${Q_{K}^{H}}$	Clinker produced on site
	and used on site to produce cement
${Q_{K}^{O}}$	Clinker produced on site
	and exported
${Q_{K}^{I}}$	Clinker imported
	and used to produce cement on site
*τ*_*I*_	Clinker Import Ratio ($\frac {{Q_{K}^{I}}}{{Q_{K}^{H}}+{Q_{K}^{I}}}$)
*τ*_*E*_	Clinker Export Ratio ($\frac {{Q_{K}^{O}}}{{Q_{K}^{H}}+{Q_{K}^{O}}}$)

In order to properly investigate the incentives involved by clinker trade, we need to distinguish:

- The clinker produced on site that is used on site to produce cement (${Q_{K}^{H}}$)
- The clinker produced on site which is exported (${Q_{K}^{O}}$).
- The clinker that is imported and used on site to produce cement (${Q_{K}^{I}}$)

The first two terms correspond to *Q*_*K*_, that intervenes in the allocation formulas of clinker OBA and hybrid OBA. The clinker ratio can be expressed as $R=\frac {{Q_{K}^{H}}+{Q_{K}^{I}}}{Q_{C}}$, where *Q*_*C*_ is the cement produced and used by the final consumer. Strictly speaking, it represents the quantity produced and released into the market.

We also define the clinker import ratio, which corresponds to the proportion of clinker in the produced cement that has been imported,$\tau _{I}= \frac {{Q_{K}^{I}}}{{Q_{K}^{H}}+{Q_{K}^{I}}}$, and the clinker export ratio, which corresponds to the proportion of clinker produced that is exported, hence not used on site to produce cement: $\tau _{E}=\frac {{Q_{K}^{O}}}{{Q_{K}^{H}}+{Q_{K}^{O}}}$.

Both *τ*_*I*_ and *τ*_*E*_ are comprised between 0 and 1. *τ*_*I*_=1 when a plant imports all of the clinker used, for example a separated grinding station. *τ*_*E*_=1 when a plant exports all of the clinker produced.^11^

We can express direct emissions of a plant with five control variables: one quantitative (*Q*_*C*_) and four qualitative (the clinker carbon intensity *I*_*K*_, the clinker ratio *R*, the clinker import ratio *τ*_*I*_ and the clinker export ratio *τ*_*E*_):

4 $$ E=I_{K} \times Q_{K}=Q_{C} \times R \times I_{K} \times \frac{1-\tau_{I}}{1-\tau_{E}}  $$

We see that beside reducing the amount of cement produced (*Q*_*C*_↘), emissions are reduced when the clinker ratio is decreased (*R*↘), or when the clinker carbon intensity is reduced (*I*_*K*_↘). Emissions also fall when more clinker is imported (*τ*_*I*_↗) or offshored, hence causing carbon and production leakage, or less clinker is exported (*τ*_*E*_↘). An adequate policy would give incentive to reduce *R* and *I*_*K*_ while staying as neutral as possible regarding *τ*_*I*_ and *τ*_*E*_.

## Key advantages of hybrid OBA

### Incentive properties

In order to study the different incentives given by the three different OBA allocation methodologies, we use a method involving two steps. First, we compute the carbon cost of cement production (emissions minus allocation, divided by cement production) for the three different allocation methods (cement, clinker and hybrid OBA). We find that the carbon cost of cement per unit of cement production depends on four variables: clinker carbon intensity (*I*_*K*_), clinker ratio (*R*), and clinker import and export ratios (*τ*_*I*_ and *τ*_*E*_) (see in the ?? Electronic Supplementary Material section 1.1). The carbon cost of cement is expressed in terms of allowances (EUAs) per ton of cement. If 1EUA=10€, then 0.10 EUA/tC translates into 1€/tC (in the rest of the paper, /tC and /tK will stand for “per ton of cement” and “per ton of clinker”).

Second, to show the influence of these variables on the carbon cost of cement (and thus the incentives for a company to modify these variables in order to reduce the carbon cost of cement), we display isocost curves.^12^ This graphical representation provides a visual understanding of incentives that cannot be obtained with calculus.

**Fig. 1:**
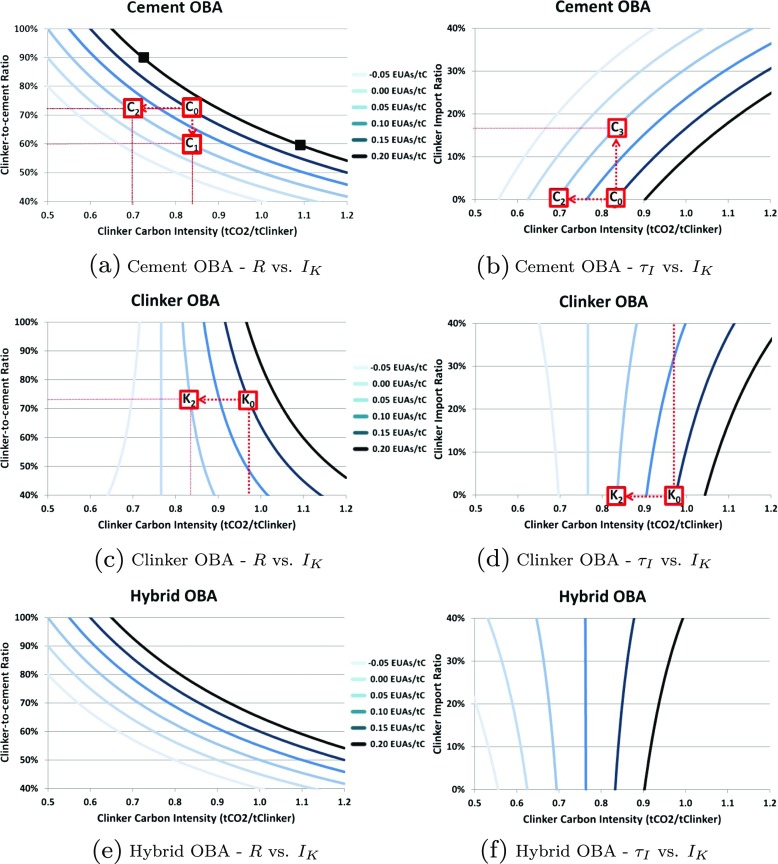
Isocost curves for the three OBA allocation schemes

The main result obtained by the analysis is that hybrid OBA combines the best elements of cement OBA and clinker OBA. That is, it gives the incentive to lower the carbon intensity of clinker and the clinker ratio, while neutralising the incentive to increase clinker imports. The explanation, which will be elaborated further, lies in the similarity between Fig. 1(e) and (a) (indicating that hybrid OBA provides similar incentive as with cement OBA to reduce the clinker ratio and the clinker carbon intensity); and between Fig. 1(f) and (d) (indicating that hybrid OBA provides similar disincentive as with clinker OBA to offshore clinker production). The rest of this section details the steps leading to these results.

In the following, we assume no clinker exports (*τ*_*E*_=0 %) in all cases. This enables focusing on the impact of the three other variables that are more relevant to leakage: clinker carbon intensity (*I*_*K*_), clinker ratio (*R*) and clinker imports (*τ*_*I*_). Figure 1 displays the isocost curves for the three types of OBA (cement, clinker, and hybrid). In the *R* vs. *I*_*K*_ graphs on the left side, *τ*_*I*_ is held constant at 0. In *τ*_*I*_ vs. *I*_*K*_ graphs on the right, *R* is held constant at 72 %, the European average in 2012.^13^

All points on the same curve represent different configurations of variables (*I*_*K*_, *R*, and *τ*_*I*_) that give the same cost of carbon per ton of cement. In Fig. 1(a) (cement OBA) for example, the two square markers on the top curve show two configurations that bear the same carbon cost: 0.20 EUA/tC. In one case, it is a high clinker-content cement with a “clean” clinker (*R*= 90 % and *I*_*K*_=0.72 tCO _2_/tK). In the other case, it is a low clinker-content cement with a “dirty” clinker (*R*= 60 % and *I*_*K*_=1.08 tCO _2_/tK).

Now let us consider a plant for which *R*=72 %, *I*_*K*_=0.835 tCO _2_/tK, *τ*_*E*_=0 %, and *τ*_*I*_=0 %. Under cement OBA, the carbon cost of cement in this case is 0.15 EUAs per ton of cement (C _0_ in Fig. 1(a) and (b)). To reduce this cost to 0.05 EUAs/tC, the firm can reduce the clinker ratio down to 60 % (C _1_), reduce clinker carbon intensity down to 0.695 tCO _2_/tK (C _2_), or increase the clinker import ratio to 17 %, which means producing 83 % of the clinker on site and buying in the rest (C _3_). While cement OBA does give incentives to reduce *R* and *I*_*K*_, at the same time it also fails to remove the incentive to abate offshoring, which would cause carbon leakage. Indeed reducing *R* to 60 % is relatively challenging in the short term but feasible, as is adjusting *I*_*K*_ to 0.695 tCO _2_/tK. Similarly, using 17 % of imported clinker requires some logistics and organisation but is achievable. For a relatively low carbon price however, the transport and other trade-related cost of importing clinker is high enough compared to the potential gain from selling unused allowances, such that the risk of carbon leakage with cement OBA small.^14^ Nonetheless the situation may change if carbon price differentials with trading partners become more important.

Compared to the diagonal curves for cement OBA, the isocost curves for clinker OBA (Fig. 1(c) and (d)) are more vertical. To illustrate the differences in incentive properties under clinker OBA, let us consider a plant for which *R*=72 %, *I*_*K*_=0.980 tCO _2_/tK and *τ*_*I*_=0 % (K _0_). Again assuming an initial carbon cost of cement of 0.15 EUA/tC, to reduce it to 0.05 EUA/tC, a firm could: decrease *R* to 25 % (K _1_, off the chart), reduce *I*_*K*_ to 0.840 tCO _2_/tK (K _2_); or increase *τ*_*I*_ to 70 % (K _3_, off the chart). Clinker OBA therefore strongly discourages carbon leakage, because the incentive to marginally increase *τ*_*I*_ is limited. A firm has to offshore a considerable share of clinker (70 %) to achieve the same carbon cost reduction, unlike with a cement OBA. Thus even though imports are not explicitly discriminated, nor is a level playing field achieved between domestic and imported clinker, clinker OBA goes a long way to discourage efforts to increase the share of imported clinker. The major downside of clinker OBA, however is that it fails to create robust incentives to reduce the clinker ratio: to gain 0.10 EUA/tC, it is necessary to reduce the clinker ratio by almost 50 percentage points, whereas under cement OBA to obtain the same result the required decrease of *R* was only 12 percentage points.

Isocost curves for hybrid OBA (Fig. 1(e) and (f)) present the same characteristics as cement OBA for the *R* vs. *I*_*K*_ diagram, and the same characteristics as clinker OBA for the *τ*_*I*_ vs. *I*_*K*_ diagram^15^. It therefore combines the efficient incentives to reduce the clinker ratio and the clinker carbon intensity as with the cement OBA, and the disincentive to import clinker as with the clinker OBA, hence solving the clinker dilemma.

In practice, each company optimises between the three abatement levers by assessing if changing the configuration of cement production is profitable depending on the allowance price and many other costs that depend on global or regional factors (e.g. energy prices or clinker price on the market) or local factors (e.g. availability of clinker substitutes or alternative fuels and transport costs). Modelling these to produce quantitative predictions of outcomes of different OBA rules (e.g. clinker ratio, clinker carbon intensity and clinker trade) would require a substantial amount of data, many assumptions and a complex optimisation model. While this is beyond the scope of this paper, nonetheless, we demonstrated that hybrid OBA has superior incentive properties over cement or clinker OBA.

### Increasing the scope of mitigation by encouraging clinker substitution across all facilities

There are two broad types of facilities producing cement:

- *“Traditional” or integrated cement plants*, producing both clinker and cement.
- *Separated grinding stations*, which do not produce clinker, but manufacture cement with clinker produced elsewhere and other clinker substitutes. Grinding stations are typically located close to sources of these substitutes.^16^

Grinding stations cannot be included in the scheme under clinker OBA (because they do not produce clinker and clinker OBA allocation is based on clinker output) but there is scope for their inclusion under cement and hybrid OBA. Including grinding stations improves the efficiency of the scheme because:

- Grinding stations play a key role in driving down cement sector emissions. Even though they do not produce clinker, their choice of clinker ratio influences clinker production volumes elsewhere.
- Excluding grinding stations under hybrid OBA could give rise to distortions, because companies would have an incentive to produce low clinker content cement in integrated plants (to receive more allowances), and produce high clinker content cement in grinding stations.

Therefore, including grinding stations expands the scope of mitigation of the cement sector, by providing economic incentives to fully leverage the mitigation potential across all facilities, maximise the use of clinker alternatives available and make low-carbon cement in grinding stations. Hybrid OBA ensures that the location of the cement production (whether it is in an integrated plant or in a grinding station) has no impact on the amount of allocation received, hence it is neutral regarding the production location.^17^

## Potential implementation issues with hybrid OBA - A case study of the EU ETS

This section explores some of the implementation issues of the hybrid OBA using the EU ETS as a case study. First we compare OBA to *ex ante* allocation, the current EU ETS allocation scheme, in Sub-section 5.1. Then we focus on specificities of the hybrid design in Sub-section 5.2.

This assessment builds on information obtained through interviews with both industry executives in all major cement companies in Europe, and policy makers (both EU and Member State level). Semi-structured telephone interviews of about one hour each were conducted with 11 individuals in 7 different EU countries between May and July 2015. The interview discussions were formulated around questions about hybrid output-based allocation, mitigation in the cement sector and allocation in the cement sector in general. While the objectivity of interviewee responses cannot be assessed, we do not expect strong strategic behaviour given the academic nature of this research which was made explicit in advance. Insights gained from the interviews informed but did not dictate our assessment.

### Implementation issues when moving from *ex ante* allocation to OBA

#### Monitoring, reporting and verification

Under OBA, authorities have to collect production data at the installation level on a yearly basis to compute allocation. For that they would have to set accounting methods, then collect, verify and process the data. In addition to upfront costs, subsequent verification costs will be proportional to the number of installations. Hence, if limited to a few sectors with a small number of installations, the on-going MRV costs are reasonably low.

Companies closely monitor production (and clinker ratio), and most plants in Europe have already been reporting much more detailed information within the Cement Sustainability Initiative. Supplementary costs for cement companies would thus be small.

Additionally, in the context of the EU ETS, moving from the current system to OBA would significantly decrease other administrative costs, specifically relating to new entrants, closure, and partial cessation.

#### Confidentiality issues around production data disclosure

Public disclosure of allocations would indirectly reveal production volumes at the installation level (basically dividing them by the benchmark value), which can clearly be considered anti-competitive information. To avoid possible collusions and disclosure of sensitive data, one way to implement OBA in practice is to base the allocation in year *t* on the output level with a lag of two years (*t*−2). For example, the allocation in 2021 would be based on the 2019 output. If there are concerns about large yearly fluctuations in output, it is also possible to smooth the allocation by taking the average of several years, for example the average of *t*−2 and *t*−3.

Interestingly, inferring production from allocation is not as straightforward with hybrid OBA as it is for cement or clinker OBA, because of the second term involving the clinker ratio.^18^

#### Impact of OBA fluctuations on the fixed cap

Unlike *ex ante* allocation, an output-based allocation applied to a sector implies that the overall sector allocation is uncapped. A number of approaches for absorbing this fluctuation within a capped ETS have been discussed, including an adjustment of the auctioning volume (in present or future years), or an ambition-neutral Allocation Supply Reserve proposed by Ecofys (2014).^19^ Whichever approach is used, it is clear that the fluctuation, and hence uncertainty, should be minimised. We show in the ?? Electronic Supplementary ?? Material (section 1.3) that the hybrid OBA is superior to either cement or clinker OBA in this regard.

### Additional issues with implementing hybrid OBA

#### Monitoring, reporting and verification

Additional MRV costs compared to clinker OBA would be very low because as mentioned, companies closely monitor not only production but also clinker imports and clinker ratio. For the regulator, verification costs are higher than in cement or clinker OBA (clinker ratio is harder to verify than production data).

In summary, net costs (deducing the gains from the simplification of new entrants, closure, and partial cessation provisions) for the administrator could possibly increase, but they would be largely outweighed by the benefits provided by hybrid OBA (see Section 6 for a summary of the benefits).

#### Heterogeneity of cement products and substitutes to be included in the clinker ratio benchmark

Whereas clinker is a highly homogeneous product, a variety of cement products exist with different technical properties mostly according to the different clinker substitutes used (Müller 2012).^20^ In addition, the availability of clinker substitutes varies considerably across regions and companies. Fly ash and slag are abundant near coal-fired power and steel plants respectively. Pozzolanas (volcanic rocks) are present only in certain regions (e.g. Italy and Greece).

Therefore, unlike clinker, setting a single benchmark for cement is less straightforward, and questions arise regarding the inclusion and exclusion of certain types of substitutes in the clinker ratio benchmark calculations. Many interviewees expressed concerns that plants without an easy access to clinker substitutes would be unjustly penalised. However, the objective of pricing carbon emissions is precisely to lower emissions - and thus the clinker ratio - at the European level. From a climate policy perspective, what matters is the amount of clinker in cement, and all constituents including fly ash from coal combustion, slag from steel production, limestone, gypsum, pozzolana, silica fume and burnt oil shale should be taken into account in the definition of the clinker ratio benchmark. Otherwise their use would not be incentivised, and this represents a carbon externality (see ?? Electronic Supplementary Material (section 1.2.1) for the incentives in the California-Quebec ETS which excludes some clinker substitutes).

#### Setting the benchmarks

In the EU ETS, the guiding rule for the definition of benchmarks is that it should be computed as the average performance in terms of kgCO _2_ per unit of output of the 10 % best performing installations (hereafter “10 % best”) (European Commission 2009). The computed value for clinker amounted to 766 kgCO _2_/tK, which is used in this paper.

With the same “10 % best” approach, cement OBA would imply a higher range of initial carbon costs across installations and would be on average costlier for the industry than a clinker OBA. This is because the distribution of cement carbon intensity is significantly more spread out than the distribution of clinker carbon intensity^21^.

In the case of hybrid OBA, combining together two “10 % best” benchmarks (“10 % best” clinker carbon intensity *multiplied* by “10 % best” clinker ratio, which is approximately 45-50 %^22^) would lead to an extremely stringent benchmark that no existing installation could meet.

A way to address this issue, while still adhering to the principle of the “10 % best”, is to use an inverse approach as follows. First, the average 10 % best performers for cement carbon intensity, *B*_*C*_ is computed. (GNR data suggests that it would be around 450 kgCO _2_/tC). Then *B*_*R*_ is computed as $B_{R}=\frac {B_{K}}{B_{C}}\simeq $ 59 %. This methodology leads to a stringency equivalent to a cement OBA.

#### Including clinker grinding stations into the scheme

The implementation of hybrid (but also cement) OBA poses the additional challenge of including separated grinding stations in the scheme. We estimate the number of such installations at about one hundred in Europe,^23^ compared to about 180-190 integrated plants (source EUTL). Some trading schemes such as the EU ETS cover only direct emissions but not indirect emissions. Thus including grinding stations with zero direct emissions could imply significant changes in the legal basis of the ETS.^24^

Including grinding stations may also face problems with perception because the allowances allocation to grinding stations would often be negative (if cement is produced with a clinker ratio higher than the benchmark). It does not pose a problem per se (allowances are not physical commodities but financial assets) but represents a conceptual innovation in emissions trading.

As the mitigation targets for the cement sector become more stringent over time, it is likely that the inclusion of grinding stations will be necessary in order to maximise abatement opportunities and meet the target. If their inclusion is not possible, a fall back option could be to implement a hybrid OBA without grinding stations, with a modified version of the California-Quebec ETS methodology (see ?? Electronic Supplementary Material), but the neutrality regarding the production location would be sacrificed.

#### Distortions in the concrete market

Further downstream from clinker and cement manufacturing is the market for concrete. Concrete is typically made by mixing aggregates with cement. Some ready-mixed concrete plants blend clinker substitutes with ordinary Portland cement (a high clinker ratio cement), instead of using cement with a low clinker ratio. By encouraging clinker substitution for cement manufacturing, hybrid OBA is likely to divert clinker substitutes that would otherwise be used in concrete plants towards cement plants, in order to gain allowances from a decrease in the clinker ratio.

Theoretically, considering concrete as the final product and applying a “clinker to concrete” ratio would eliminate this inefficiency. But concrete plants are significantly more numerous than cement plants, hence it would entail heavy administrative costs compared to small gains (this practice being marginal, the avoided distortion would be small).

## Conclusion

This paper focuses on OBA in emissions trading systems, one of the main options discussed to offer carbon leakage protection. Specifically we evaluate three OBA design options in the context of the cement sector by developing an analytical model of sector emissions as a function of different mitigation levers. This enables us to precisely assess the impact that changes in the technical parameters have on carbon costs, and evaluate the trade-off between the mitigation levers under each design. This assessment highlights how the design choice has an important effect on mitigation and trade incentives. We propose a specific design - the hybrid OBA - and show that it out-performs both alternative options - cement or clinker OBA - and solves the clinker dilemma, a prominent issue with output-based allocation in this sector. Hybrid OBA is a clinker OBA modified with an allowances bonus-penalty, depending on the clinker ratio (formula in Eq. 3). It has a number of advantages over alternative OBA designs and over *ex ante* allocation methods.

Relative to *ex ante* allocation methods, hybrid OBA:

- Better ensures the prevention of carbon leakage.
- Reduces the risk of excess allocation and associated over-allocation profits.
- Without excess allocation, the benchmarks under OBA provide a focal point for energy efficiency improvements if set at sufficiently ambitious levels.
- Removes the perverse incentives that occur in some *ex ante* allocation designs, such as the incentive to produce excess volumes of clinker in order to obtain more emission allowances (Branger et al. 2015).

Relative to clinker or cement OBA, hybrid OBA:

- Provides incentives which are aligned with the mitigation options available to this sector in the short to medium term i.e. it encourages reducing the clinker carbon intensity and the clinker ratio and at the same time discourages the offshoring of clinker production (see Section 4.1).
- Expands the scope of mitigation by extending the ETS to include downstream installations (grinding plants) and ensuring a system which is neutral to the production location. For example, producing lower-carbon cement in grinding stations with cheap access to clinker substitutes is not penalised but encouraged.
- Implies less fluctuations of the total volume of allowances to the sector (see ?? Electronic ?? Supplementary Material).

We argued that the two common objections to hybrid OBA – administrative complexity and the geographical heterogeneity of clinker substitutes which give an advantage to low-carbon cement producers with access to cheap clinker substitutes - do not represent major impediments (see Sections 5.2.1 and 5.2.2). The inclusion of grinding plants into the ETS may be difficult legally, politically and administratively, but fall back options exist (see Section 5.2.4). The two well-known fundamental shortcomings of OBA, however, cannot be overcome without additional policies:

- It does not give enough incentive for demand substitution, as it blocks carbon costs from being reflected in final goods prices.
- It does not promote radical technological innovation in the sector.

Both shortcomings are related because without a carbon price signal, cement consumers tend to resist against new, untested products, which in turn acts as a barrier for cement producers to reduce the clinker content of cement.^25^ These are important impediments to the long-term decarbonisation of the cement sector, and we therefore argue that hybrid OBA should be seen as a short or medium term solution, while transitioning to a more robust leakage prevention regime. An alternative option is to combine OBA with a consumption levy as proposed in Neuhoff et al. (2014), to help stimulate demand and production of low-carbon alternatives. In cement, such a consumption charge could be applied to clinker (in addition to clinker OBA), or to cement (with possibly lower administrative costs).

In the cement sector, there is an emissions gap between what technology roadmaps enable (IEA 2009), and the emissions trajectories needed to avoid detrimental climate change. Providing free allocation in proportion to output to this sector may certainly not promote the radical innovation required to close this emissions gap in the long term, but auctioning alone may not be sufficient either. Emissions trading schemes must then be completed with ambitious policies to correct market failures. Significant public investment will be necessary to bring about carbon capture and storage (CCS) demonstration plants. To encourage more efficient use of cement in buildings, or the development of new low-carbon materials, public funding will likely be necessary as well as changes to building regulations and standards. At the same time, reforming the ETS will also play an important role. For example a price floor rising over time, as in the California-Quebec ETS, certainly gives the right signal to investors in new low-carbon building materials.

## Electronic supplementary material

Below is the link to the electronic supplementary material.

(PDF 659 KB)

## Footnotes

1
^1^ Some *ex post* adjustment is also possible in the EU ETS for low activity installations, and also in the Shanghai and Shenzhen pilot schemes (ICAP 2015).

2
^2^ This study attributes the lack of marked emissions intensity improvements in the EU ETS phase 1–3 to be in part driven by the combination of low carbon prices and over-allocation of free allowances. In addition, contrary to theory, this study finds that companies respond differently to carbon prices if they represent an opportunity cost rather than a real cost, for example due to the prevalence of cost plus pricing strategies adopted within companies.

3
^3^ This paper shows that in conditions of low-demand, partial cessation rules in free allocation methodology (free allocation is halved if production drops below 50 % of historical output) can provide incentives for regulated companies to artificially increase production volumes in order to maintain maximum free allocation

4
^4^ It is worth noting that the decision on whether to apply the benchmark on clinker or cement can also occur in *ex ante* allocation (clinker was finally chosen in the EU ETS). But in that case, it only gives rise to *distributional* implications. For the EU ETS, choosing a cement benchmark would have involved distributing more allowances to companies that historically had a low clinker ratio (the proportion of clinker in cement), but the incentive to lower the clinker ratio would have remained identical.With *ex post* OBA however, we will see that such a choice has *incentive* implications: the incentives to lower the clinker ratio are modified by this decision.

5
^5^ See Electronic Supplementary Material for a study of incentives in California-Quebec and New Zealand ETS. Another difference between our proposed hybrid OBA and the California-Quebec and New Zealand ETS is that we include downstream installations (grinding stations). The benefits of their inclusion are described in Section 4.2.

6
^6^ In 2012-2013 it represented 6 % of EU ETS emissions, steel standing for 7 % (source: own computation from the EUTL, the EU ETS registry).

7
^7^ While in theory benchmarking under *ex ante* allocation also incites efficiency improvements, in practice the incentive is dampened if installations receive more free allocation than they need. This can be explained by the observation in the EU ETS that firms do not respond to opportunity costs the same way as they respond to actual costs (Branger et al. 2015).

8
^8^ They can only be stored (with CCS), or avoided, through reduced production or alternative cements which are at the development stage, such as Novacem (based on magnesium silicates rather than limestone), Calera or Geopolymer (Schneider et al. 2011).

9
^9^ To decrease the clinker carbon intensity, one can decrease the energy intensity of the kiln, or use alternative fuels which are less carbon intensive (like biomass). For a detailed analysis of the abatement levers and the decomposition of emissions in the European cement industry, see Branger and Quirion (2015).

10
^10^ These are either by-products of other industries or natural materials, and have a much lower carbon intensity than clinker.

11
^11^ There is only one such plant in Europe. Virtually all clinker facilities manufacture cement as well (Ref. interviews with industry executives).

12
^12^ This method is an application of the isocost curves used in standard microeconomic theory e.g. Varian (2009).

13
^13^ The parameters values cover a wide range: 40 % to 100 % for the clinker ratio *R*, 0 % to 40 % for the clinker import ratio *τ*_*I*_, and 0.5 to 1.2 tCO _2_/tK for the clinker carbon intensity *I*_*K*_. We use clinker carbon intensity benchmark values *B*_*K*_=766 kgCO _2_/tK (like in the EU ETS), cement carbon intensity benchmark *B*_*C*_=450 kgCO _2_/tC (same methodology with the Getting the Number Rights (GNR) data (WBCSD 2009)) and clinker-ratio $B_{R}=\frac {B_{K}}{B_{C}}$=59 % (the hybrid and cement OBAs then have the same stringency, see Section 5.2.3 for the determination of the benchmark values).

14
^14^ Indeed, with the current EU ETS allocation rules (mostly independent of production), clinker offshoring allows to benefit from the full opportunity cost of allowances, but econometric studies have not revealed any statistical evidence of an impact of carbon price on clinker net imports (Branger et al. 2013).

15
^15^ The left shift of the isocost curves between Fig. 1(d) and (f) is because for *τ*_*I*_ vs. *I*_*K*_ diagrams, *R*=75 % (>*B*_*R*_), so for a given *τ*_*I*_ and *I*_*K*_, hybrid OBA is more costly. It would be the opposite if *R* was lower than the clinker benchmark.

16
^16^ The bulk of clinker used in a typical grinding station comes from the closest clinker plant owned by the same company as the cheapest source of clinker, but this is changing over time as inter-company clinker trade has been increasing in recent years. The majority of grinding stations are owned by companies producing clinker, although there is a small number of independent grinding stations belonging to companies only buying clinker in the market. The major determinants of the location of grinding stations include the availability of substitutes, access to port facilities, and location of demand.

17
^17^ As a simple example, consider a company producing *Q*_*C*_ cement with a clinker ratio *R* (so we have *Q*_*K*_ = *R*×*Q*_*C*_). An integrated plant would receive the allocation $A_{Integrated Plant}=B_{K} \times Q_{K} + B_{K}(B_{R}-R)\times Q_{C}=B_{K} \times Q_{K} \times \frac {B_{R}}{R}$. In case of production in two separated installations, the clinker-producing plant would receive *A*_*C**l**i**n**k**e**r**P**l**a**n**t*_ = *B*_*K*_×*Q*_*K*_ (first “part” of *A*_*I**n**t**e**g**r**a**t**e**d**P**l**a**n**t*_) and the grinding station would receive *A*_*G**r**i**n**d**i**n**g**S**t**a**t**i**o**n*_ = *B*_*K*_(*B*_*R*_−*R*)×*Q*_*C*_ (second “part” of *A*_*I**n**t**e**g**r**a**t**e**d**P**l**a**n**t*_) so the total allocation is similar as if it was made by an integrated plant. We can verify that it is still the case in a more complex configuration with imports, exports, incomplete transfer of clinker to the grinding station, and production of cement on both sites.

18
^18^ Excluding the cross sectoral correction factor, an allocation of 0.5 MEUAs can be explained by a cement production of 0.92 Mt (with *R*=95 %) or 1.20Mt (with *R*=50 %).

19
^19^ A tiered approach could also be used, where carbon leakage sectors are grouped into high, medium or low exposure levels; the highly exposed sectors being guaranteed to receive free allowances proportional to 100 % output (multiplied by benchmark), and the “slack” taken up by adjusting the free allocation to the medium and low exposure sectors.

20
^20^ The European Cement Standard EN 197-1 differentiates eight types of Portland cement, and three types of non-Portland cement depending on cement composition.

21
^21^ GNR (WBCSD 2009) provides distribution graphs of these two variables (variable 59cDG for clinker and 59CDGWct for cement). In 2013, the weighted average and standard deviation were respectively 830 kgCO _2_/tK and 66 kgCO _2_/tK for clinker, and 627 kgCO _2_/tC and 175 kgCO _2_/tC for cement. Furthermore, the cement distribution graph is not made with installations contrary to the clinker distribution graph, but with national companies values (combining all clinker and grinding installations of one company in a country). This “flattens” the curve with an averaging effect, so the dispersion would be significantly larger if the curve was computed with installations.

22
^22^ Variable 92DGW (distribution of clinker ratio for cement) in GNR.

23
^23^ Source: own computation from interviews of industry executives. Between 2006 and 2008, there were 90 grinding stations in the EU http://eippcb.jrc.ec.europa.eu/reference/BREF/CLM_30042013_DEF.pdf

24
^24^ In the EU ETS this requires fundamental changes to the EU ETS Directive. Grinding stations are also excluded from the Integrated Pollution Prevention and Control (IPPC) Directive.

25
^25^ Clinker substitution can change the technical properties of cement, including strength and sulphate resistance, as well as colour and workability.
