# Supplementary material for: Solving the clinker dilemma with hybrid output-based allocation
Source: Clim Change. 2017 Jan 18;140(3):483–501. doi: 10.1007/s10584-016-1884-x (PMC7175600; doi:10.1007/s10584-016-1884-x)
Supplement: Supplementary file 1 — (PDF 659 KB) [file 10584_2016_1884_MOESM1_ESM.pdf]

1. Electronic Supplementary Material

1.1. Isocarboncosts curves computation

Using Equation (4), we can compute the marginal cost of carbon in cement, which is equal in this case to the mean cost of carbon, $\dfrac{E-A}{Q_C}$, for the three allocation methodologies: cement; clinker and hybrid OBA.

First, the allocations formulas for the clinker (Equation (2)) and the hybrid OBA (Equation (3)) can be re-written in order to ease the comparison with emissions from Equation (4)).

$$\begin{aligned} A_K &= B_K \times Q_K \\ &= B_K \times [Q_K^H + Q_K^O] \\ &= B_K \times R \times \frac{1-\tau_I}{1-\tau_E} \times Q_C \end{aligned} \tag{1}$$

$$\begin{aligned} A_{Hyb} &= B_K \times Q_K + B_K(B_R - R) \times Q_C \\ &= B_K \times [Q_K^H + Q_K^O] + B_K(B_R - R) \times Q_C \\ &= [B_K \times R \times \frac{1-\tau_I}{1-\tau_E} + B_K(B_R - R)] \times Q_C \end{aligned} \tag{2}$$

The marginal cost curves are:

$$MC_C = \frac{1-\tau_I}{1-\tau_E} \times R \times I_K - B_C \tag{3}$$

$$MC_K = \frac{1-\tau_I}{1-\tau_E} \times R \times [I_K - B_K] \tag{4}$$

$$MC_{Hyb} = \frac{1-\tau_I}{1-\tau_E} \times R \times [I_K - B_K] + B_K(R - B_R) \tag{5}$$

The equation of isocarboncosts curves are:

$$\begin{aligned} MC_C = MC^0 &\iff R = (1-\tau_E)\frac{MC^0 + B_C}{(1-\tau_I)I_K} \\ &\iff \tau_I = 1 - (1-\tau_E)\frac{MC^0 + B_C}{I_K R} \end{aligned} \tag{6}$$

$$\begin{aligned} MC_K = MC^0 &\iff R = (1-\tau_E)\frac{MC^0}{(1-\tau_I)(I_K - B_K)} \\ &\iff \tau_I = 1 - (1-\tau_E)\frac{MC^0}{(I_K - B_K)R} \end{aligned} \tag{7}$$

$$\begin{aligned} MC_{Hyb} = MC^0 &\iff R = (1-\tau_E)\frac{MC^0 + B_K B_R}{(1-\tau_I)I_K + (\tau_E - \tau_I)B_K} \\ &\iff \tau_I = 1 - (1-\tau_E)\frac{MC^0 + B_K(B_R - R)}{(I_K - B_K)R} \end{aligned} \tag{8}$$

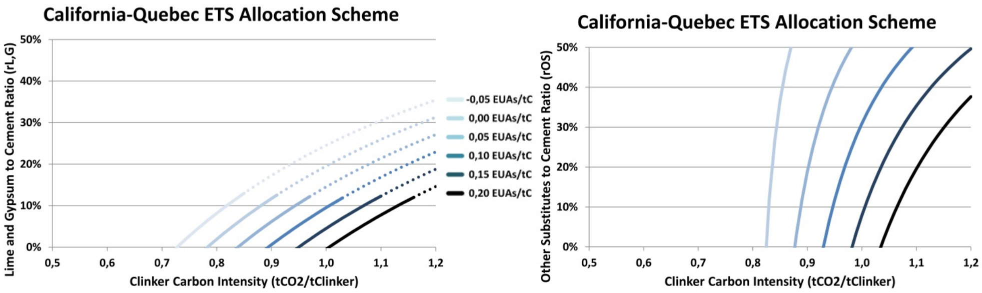

(a) California-Quebec - $r_{L,G}$ vs. I_K (b) California-Quebec - r_{OS} vs. I_K

Figure 1: Isocarboncosts curves for the California-Quebec ETS allocation scheme

1.2. Incentives for cement under other OBA designs

1.2.1. California-Quebec

In the California-Quebec ETS, which includes about 13 integrated cement plants, the output is the "adjusted clinker and mineral additives produced", defined as "Clinker Produced × (1 + (Limestone and Gypsum Consumed)/Clinker Consumed))" (California Air Resources Board, 2011):

$$A_{Cali} = B_{C,Cali} \times Q_K \times (1 + \frac{Q_{L,G}}{Q_K^I + Q_K^H}) \tag{9}$$

$Q_{L,G}$ being the quantity of limestone and gypsum consumed. The value of the benchmark is B_{Cali} =0.783 tCO_2 (less stringent than the EU ETS benchmark).

The original analytical framework has to be modified to analyse the incentives. In addition to limestone and gypsum $Q_{L,G}$, other substitutes Q_{OS} such as slag or fly ash can be used. We have then $Q_C = Q_K^I + Q_K^H + Q_{L,G} + Q_{OS}$. Noting $r_{L,G} = \frac{Q_{L,G}}{Q_C}$, the proportion of lime and gypsum in cement, and $r_{OS} = \frac{Q_{OS}}{Q_C}$, the proportion of other substitutes in cement, we have $R + r_{L,G} + r_{OS} = 1$. The substitution potential for lime and gypsum (reducing R by about 5 to 10 percentage points) is by far less important than the one for other substitutes (reducing R by more than 50 percentage points).

Rewriting (9), we have

$$\begin{aligned} A_{Cali} &= B_{Cali} \times Q_K \times (1 + \frac{r_{L,G}}{R}) \\ &= B_{Cali} \times Q_C \times \frac{1-\tau_I}{1-\tau_E} \times (1 - r_{OS}) \end{aligned} \tag{10}$$

Then the marginal cost of carbon in cement is equal to:

$$MC_{Cali} = \frac{1-\tau_I}{1-\tau_E} \times [I_K - B_{Cali} - r_{OS}(I_K - B_{Cali}) - r_{L,G} I_K] \tag{11}$$

Isocarboncost curves cannot be displayed in the same way as in section 4.1 because there is an additional variable. Figure 1 shows isocarboncost curves for the California-Quebec ETS in $r_{L,G}$ vs. I_K and r_{OS} vs. I_K diagrams (τ_I vs. I_K diagrams is similar to a clinker benchmark, that is importing clinker is disincentivised). We see that clinker substitution is incentivised, but only for gypsum and lime. However, the latter is limited to a few percentage points, so the overall incentive for clinker substitution remains low.

1.2.2. New Zealand

The NZ ETS, which includes two integrated cement plants, has two benchmarks[1]: one for clinker ($B_{K,NZ}$=0.9392 tCO_2/tK), and one for milling clinker into cement (B_M = 0.02266 tCO_2/tC), which applies to cement made with clinker produced by the plant (not imported).

[1] http://www.mfe.govt.nz/more/cabinet-papers-and-related-material-search/cabinet-papers/climate-change/climate-change-and-19

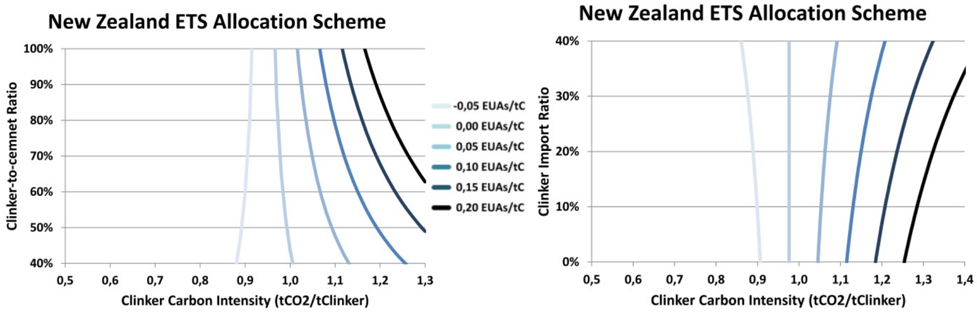

(a) New Zealand - $r_{L,G}$ vs. I_K

(b) New Zealand - r_{OS} vs. I_K

Figure 2: Isocarboncosts curves in the New Zealand ETS allocation scheme

With our notations, we define cement milled from own clinker as $(1-\tau_I)Q_C$, that is, if 10% of the clinker is imported, then only 90% of the cement produced is considered milled from own clinker. The allocation is then:

$$A_{NZ} = B_{K,NZ} \times Q_K + B_M \times (1-\tau_I)Q_C \tag{12}$$

The marginal cost of carbon is equal to:

$$MC_{NZ} = \frac{1-\tau_I}{1-\tau_E} \times [R(I_K - B_{K,NZ}) - B_M(1-\tau_E)] \tag{13}$$

Figure 2 shows isocarboncost curves for the New Zealand scheme, which are very similar to a clinker OBA (except there is a shift on the right because benchmarks are much less stringent than in our study).

1.2.3. Fall-Back option

If we modify the California-Quebec formula by including not only limestone and gypsum, but all substitutes to clinker, we have (FB stands for fall-back):

$$A_{FB} = B_C \times \frac{Q_K}{R} \tag{14}$$

Similarly to the California-Quebec OBA, this allocation methodology can only apply to facilities producing clinker. B_C is a cement benchmark, which can be identical to the one used in cement OBA.

The marginal cost of carbon is equal to:

$$MC_{FB} = \frac{1-\tau_I}{1-\tau_E} \times [R \times I_K - B_C] \tag{15}$$

Figure 3 shows that isocarboncost curves are similar to the hybrid OBA. Hence, the fall-back option gives the appropriate incentives: reducing the clinker ratio but not offshoring of clinker production.

However, such a system is not neutral regarding the production location. It disincentivises producing clinker in one installation and using it to manufacture cement in another installation (separated grinding station or other plant). Let us consider the same example as in section 4.2 of a company producing Q_C cement with a clinker ratio R (so we have $Q_K = R \times Q_C$). An integrated plant would receive the allocation $A_{IntegratedPlant} = B_C \times \frac{Q_K}{R}$, while in case of production in two separated installations, the “clinker-only” plant would receive $A_{ClinkerPlant} = B_C \times Q_K$ and the other installation would not receive any allocation (because it would not produce clinker). The total allocation would then be equal to $B_C \times Q_K < B_C \times \frac{Q_K}{R}$.

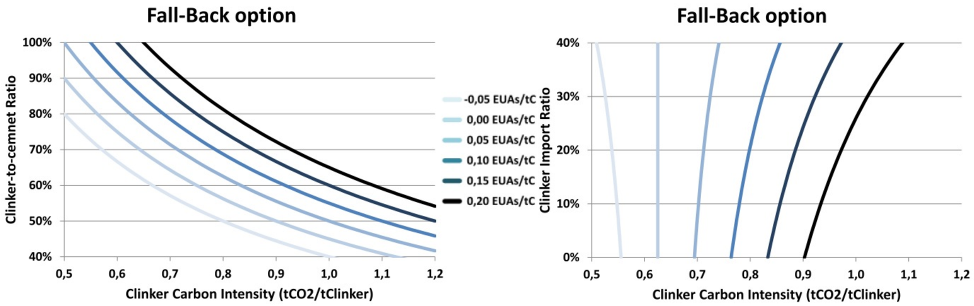

(a) Fallback Benchmark - R vs. I_K (b) Fallback Benchmark - τ_I vs. I_K

Figure 3: Isocarboncosts curves for the fall-back option

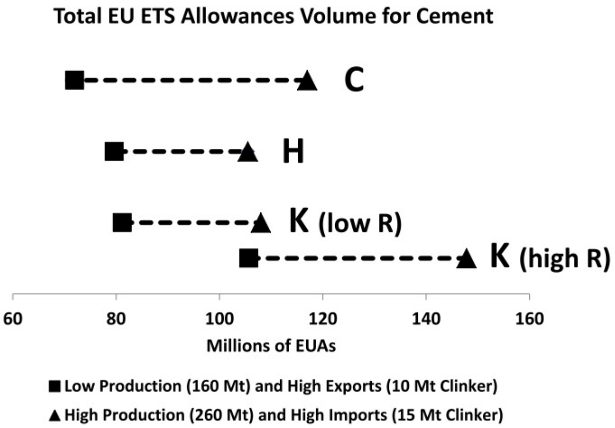

Figure 4: Total EU ETS allowances volume for cement under a cement OBA (C), hybrid OBA (H) and clinker OBA (K)(in millions of EUAs).

1.3. Minimising fluctuation of allocation volume

Another key advantage with the hybrid OBA is that compared to cement or clinker OBA, fluctuations of the total volume of allowances to the sector are minimised. To illustrate this, Figure 4 displays the level of the cement sector allocation for the EU ETS, under different OBA benchmarks: cement (C), hybrid (H) and clinker (K). We compare the case with high production levels and high imports (triangle marker) against the case with low production and high exports (square marker). For clinker, we also contrast scenarios with high and low average clinker ratios[2]). The orders of magnitude of production and trade are intended to be realistic.[3]

Changes in the total volume of free allocation to the sector are first and foremost driven by output variability. By definition with output-based allocation, variability in output levels induce changes in total allocation. However, we find that the magnitude of this variability in total allocation is lower in hybrid OBA:

[2]The clinker ratio has no impact whatsoever on the total allocation for cement OBA because the latter only depends on cement production. It is also the case for hybrid OBA, which could seem surprising as the clinker ratio is used to determine the allocation. The reason is as follows. For a given cement production (and a given clinker trade), when the clinker ratio decreases, less clinker has to be produced, so less allowances are given in proportion of clinker output (the first part of hybrid OBA allocation). But this allowances loss is *exactly compensated* by the "allowances bonus" rewarding the decrease of the clinker ratio (the second part of hybrid OBA allocation).

[3]High production corresponds to pre-crisis production at the EU ETS level. Low production corresponds to 2013 production. Clinker net imports in the EU 27 were at 14.1 Mt in 2007 (their highest just before the crisis) and clinker net exports were at 7.6 Mt in 2013. Source: Eurostat: (cement clinker: 252310) http://epp.eurostat.ec.europa.eu/newxtweb/setupdimselection.do. Clinker imports may be higher in cement OBA, but their magnitude have no impact on the total allocation (which only depends on the quantity of cement produced).

[80-106], compared to cement and clinker OBAs with [72-117] and [81,148] respectively.[4] Because typically the overall cap of the whole emissions trading system is fixed in line with the region's mitigation targets, the reduced uncertainty about the total allowances volume is clearly a desirable feature for an output-based allocation.

References

California Air Resources Board (2011). Cap-and-Trade Regulation Discussion Draft. Appendix B: Development of Product Benchmarks for Allowance Allocation. Technical report.

[4]The total allocation in clinker OBA is significantly impacted by a change in the clinker ratio. As we explained, under clinker OBA the incentive to reduce R is very low, so "high R" is the most probable case. The magnitude would then be reduced to [106,148], which is still larger than in Hybrid OBA.
